# Correction: Ribeiro et al. Liposomal Formulations of a New Zinc(II) Complex Exhibiting High Therapeutic Potential in a Murine Colon Cancer Model. *Int. J. Mol. Sci.* 2022, *23*, 6728

**DOI:** 10.3390/ijms27104339

**Published:** 2026-05-13

**Authors:** Nádia Ribeiro, Melissa Albino, Andreia Ferreira, Cristina Escrevente, Duarte C. Barral, João Costa Pessoa, Catarina Pinto Reis, Maria Manuela Gaspar, Isabel Correia

**Affiliations:** 1 Centro Química Estrutural, Departamento de Engenharia Química, Instituto Superior Técnico, Universidade de Lisboa, 1049-001 Lisboa, Portugaljoao.pessoa@tecnico.ulisboa.pt (J.C.P.); 2 Research Institute for Medicines (iMed.ULisboa), Faculty of Pharmacy, Universidade de Lisboa, 1649-003 Lisboa, Portugalcatarinareis@ff.ulisboa.pt (C.P.R.); 3 iNOVA4Health, NOVA Medical School (NMS), Faculdade de Ciências Médicas (FCM), Universidade Nova de Lisboa, 1169-056 Lisboa, Portugalduarte.barral@nms.unl.pt (D.C.B.); 4 IBEB, Faculdade de Ciências, Universidade de Lisboa, Campo Grande, 1749-016 Lisboa, Portugal

## Figure Legend

In the original publication [1], a duplication of controls in Figure 1B was done. The authors tried to look on the computers where these assays had been performed for additional figures. Unfortunately, this was not possible. Given this, The authors decision is to remove Figure 1B completely from the publication that corresponds to qualitative results. Furthermore, Figure 1A presented the quantitative results of LDH assay, showing the cytotoxic effect of metal-based complexes after incubation in murine (CT-26) and human (HCT-116) colon cancer cell lines, which is quite low in comparison with the negative controls in this 3D in vitro model. The corrected Figure 1 appears below, as well as the revised legend.

Regarding Figure 4, bright field images do not have optical sections like confocal ones and, therefore, there is only one image per condition. Thus, the duplication was only done to align the bright field with the confocal images and there was no intention of using the same image to illustrate different conditions or results. The authors have corrected the figure to leave just one bright field image for each spheroid. The legend of Figure 4 has not been changed. Nevertheless, it was included in this document. The corrected Figure 4 appears below, as well as its legend.

## Text Correction

Due to the removal of Figure 1B, where it was written (Figure 1A) is changed to (Figure 1); the sentence “Moreover, the obtained spheroids’ images (Figure 1B) also revealed solubility problems when higher concentrations of the zinc complexes were used, particularly in the case of [ZnL2]” should be removed.

The authors state that the scientific conclusions are unaffected. This correction was approved by the Academic Editor. The original publication has also been updated.

## Figures and Tables

**Figure 1:**
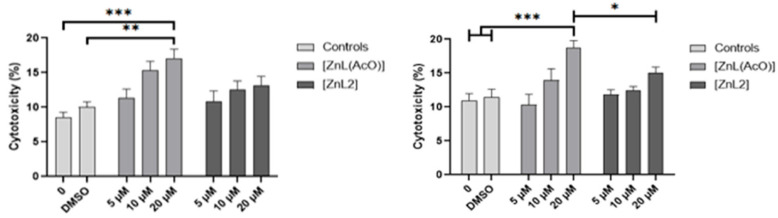
Cytotoxicity effect of zinc complexes [ZnL(AcO)] and [ZnL2] towards CT-26 and HCT-116 spheroids. Results correspond to cellular toxicity expressed as mean percentage (%) ± SD of three independent experiments with three replicates each after 72 h incubation with different metal-complex concentrations (5, 10, and 20 µM) in their free forms (LDH assay) (* *p* < 0.05 vs. C8 at 20 µM, ** *p* < 0.01, *** *p* < 0.001 vs. controls (0/ DMSO)).

**Figure 4:**
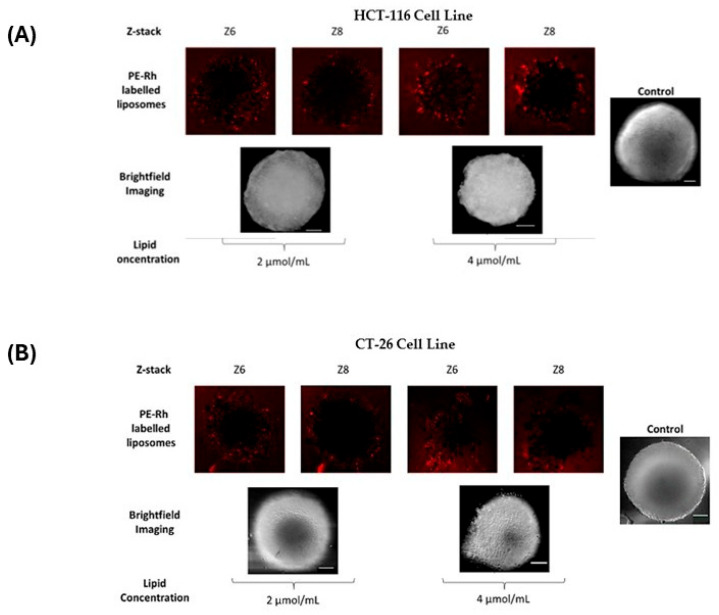
Liposome internalization in CRC cell line spheroids after 120 h of incubation with 2 and 4 µmol/mL concentrations of [ZnL(AcO)]. Z-stack images from the periphery to the center of the spheroid, taken by confocal microscopy, show penetration of Rh-labelled liposomes in CRC human and murine cell line HCT-116 (**A**) and CT-26 (**B**) spheroids after 120 h of incubation. The red color indicates the presence of Rh-labelled liposomes. Scale bar = 100 μM.
