# Supplementary material for: Liposomal Formulations of a New Zinc(II) Complex Exhibiting High Therapeutic Potential in a Murine Colon Cancer Model
Source: Int J Mol Sci. 2022 Jun 16;23(12):6728. doi: 10.3390/ijms23126728 (PMC9223407; doi:10.3390/ijms23126728)
Supplement: Supplementary file 1 [file ijms-23-06728-s001.zip › ijms-1763777-supplementary.pdf]

International Journal of *Molecular Sciences*

MDPI

Supplemental Material

Liposomal formulations of a new zinc(II) complex exhibiting high therapeutic potential in a murine colon cancer model

Nádia Ribeiro [1,†], Melissa Albino [2,†], Andreia Ferreira [3], Cristina Escrevente [3], Duarte C. Barral [3], João Costa Pessoa [1], Catarina Pinto Reis [2,4], Maria Manuela Gaspar [2,*,‡] and Isabel Correia [1,*,‡]

[1] Centro Química Estrutural, Departamento de Engenharia Química, Instituto Superior Técnico, Universidade de Lisboa, 1049-001 Lisboa, Portugal; nadia.ribeiro@tecnico.ulisboa.pt (N.R.); joao.pessoa@tecnico.ulisboa.pt (J.C.P.)
[2] Research Institute for Medicines (iMed.Ulisboa), Faculty of Pharmacy, Universidade de Lisboa, 1649-003 Lisboa, Portugal; melissateixeira@campus.ul.pt (M.A.); catarinareis@ff.ulisboa.pt (C.P.R.)
[3] iNOVA4Health, NOVA Medical School (NMS), Faculdade de Ciências Médicas (FCM), Universidade Nova de Lisboa, 1169-056 Lisboa, Portugal; andreia.ferreira@nms.unl.pt (A.F.); cristina.escrevente@nms.unl.pt (C.E.); duarte.barral@nms.unl.pt (D.C.B.)
[4] IBEB, Faculdade de Ciências, Universidade de Lisboa, Campo Grande, 1749-016 Lisboa, Portugal
* Correspondence: mgaspar@ff.ulisboa.pt (M.M.G.); icorreia@tecnico.ulisboa.pt (I.C.)
† These authors contributed equally to this work.
‡ These authors contributed equally to this work.

Contents

S1 - Characterization of the Zn(II) complexes 1
S2 - Studies of interactions with BSA 3
S3 - Studies of interactions with ctDNA 7
S4 – Cell death and cell cycle analysis 9

S1 - Characterization of the Zn(II) complexes

Despite several attempts, it was not possible to obtain single crystals suitable for X-ray diffraction. Thus, spectroscopic features of the coordinating groups were used to complement the elemental analysis and mass spectrometry results in order to provide a description of the Zn(II) complexes synthetized in this work. The main results are summarized in Table S1, while Figures S1, S2 and S3 show the spectra for FTIR, UV-Vis absorption and ^{1}H NMR, respectively.

Table S1. Hydroxyl and imine functions FTIR (in cm^{-1}) and NMR signals (in ppm) in the ligand precursor and Zn(II) complexes.

	ν_{OH} (cm^{-1})	$\nu_{C=N}$ (cm^{-1})	δ_H^{OH} (ppm)	$\delta_H^{C=N}$ (ppm)	$\delta_C^{C=N}$ (ppm)
**HL**	3411	1621	9.82	8.34	144.1
**[ZnL(AcO)]**	—	1610	—	9.90	144.9
**[ZnL$_2$]**	3411	1561	9.79	8.34	143.6

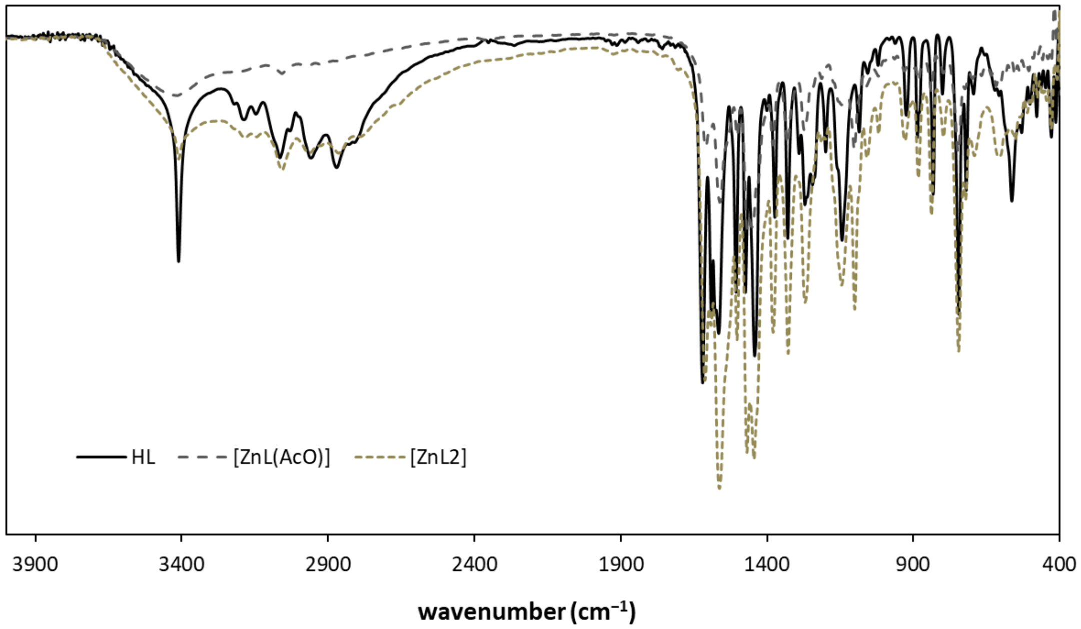

Figure S1. Infra-Red spectra of HL, **[ZnL(AcO)]** and **[ZnL_2]** obtained as KBr pellets.

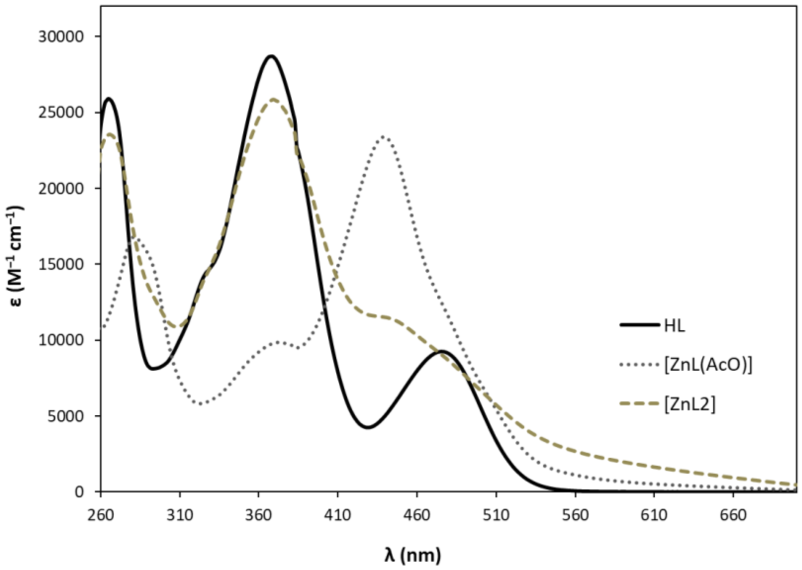

Figure S2. UV-Vis absorption spectra of HL, **[ZnL(AcO)]** and **[ZnL_2]** in DMSO at *ca.* 35 μM.

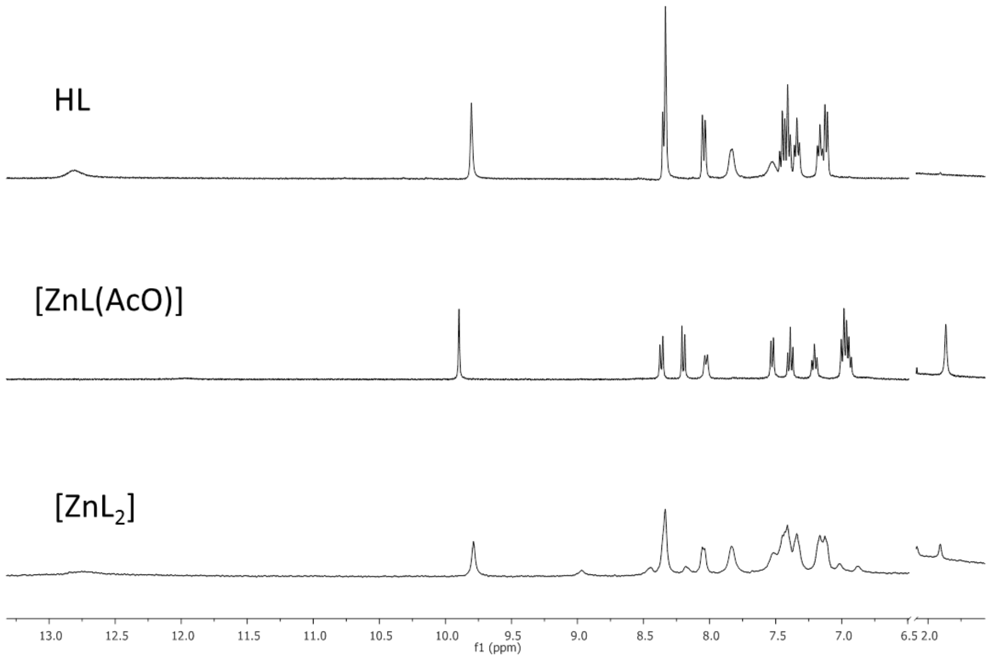

Figure S3. ^{1}H NMR spectra of HL, **[ZnL(AcO)]** and **[ZnL$_2$]** in DMSO_d_6 in a 400 MHz apparatus.

S2 - Studies of interactions with BSA

A compound to be used as a drug has to possess water solubility and stability to some extent. The zinc complexes synthetized in this work present very low water solubility and, furthermore, even when dissolved in an organic solvent, they start to precipitate when in contact with an aqueous medium. This effect can be observed in the UV-Vis absorption spectra in Figure S4.

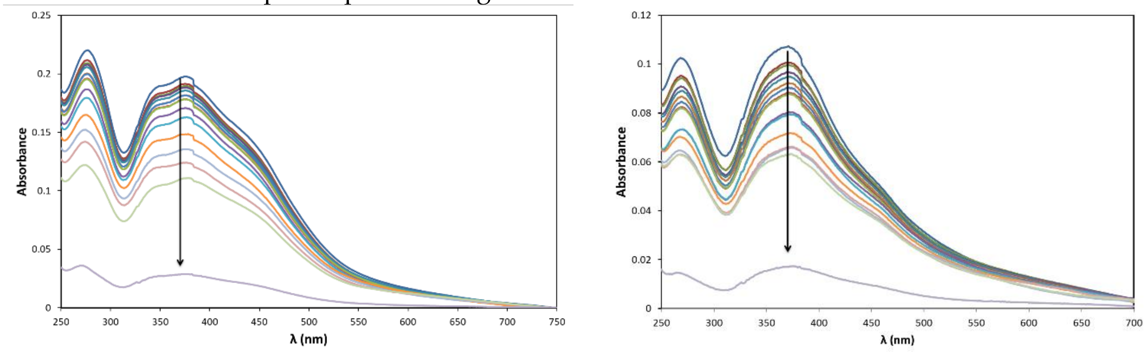

Figure S4. UV-Vis spectrum of a solution **[ZnL(AcO)]** = 28 μM (left) and **[ZnL$_2$]** = 14 μM (right), in 0.6% DMSO/ PBS buffer (pH 7.4; 0.01 M phosphate; 0.138 M NaCl; 0.0027 M KCl); arrows show the changes occurring during 24 h of experiment.

A way to circumvent this effect is to protect them in a hydrophobic environment. When traveling inside a living organism, several of these exogenous compounds bind to carrier proteins, such as albumins. In fact, as we can see in Figure S5, when the complexes are bound to BSA there is a substantial increase in their stability and solubility in the aqueous medium.

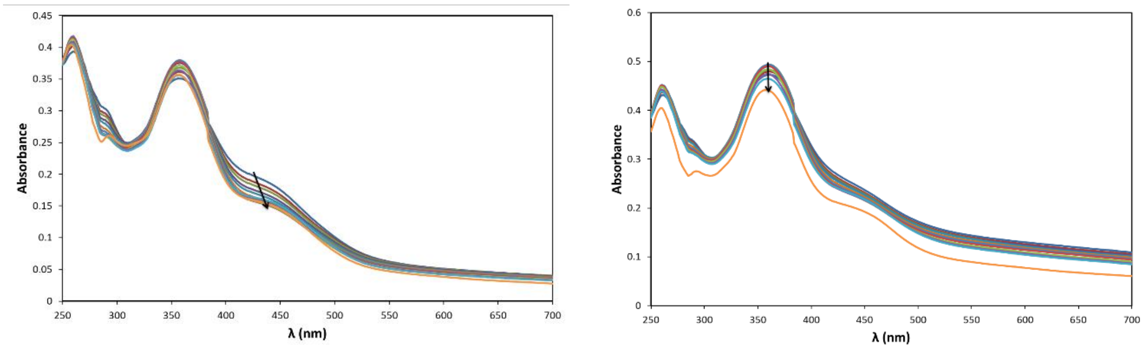

Figure S5. UV-Vis spectrum of a solution **[ZnL(AcO)]** = 24 μM (left) and **[ZnL₂]** = 22 μM (right) with equimolar concentration of BSA, in 0.6% DMSO/ PBS buffer (pH 7.4; 0.01 M phosphate; 0.138 M NaCl; 0.0027 M KCl); arrows show the changes occurring during 24 h of experiment.

Spectrophotometric techniques are easy and accessible methods to perform interaction studies with proteins and calculated the binding constant. Bovine serum albumin (BSA) exhibits fluorescence emission due to the presence of tryptophan (Trp) residues in its structure. When a substance interacts with BSA in a way that affects the Trp surroundings, a decrease in the intensity of the fluorescence emission is observed, in a process named quenching. The process can be analysed with the Stern-Volmer equation, which is related to the efficiency of the quenching process, providing clues on the type of involved phenomenon [1]:

$$I_0/I = 1 + k_q\tau_0[Q] = 1 + K_{SV}[Q]$$

here I_0 and I are the fluorescence intensities in the absence and presence of quencher, respectively, corrected for reabsorption and inner filter effects [2,3], k_q is the bimolecular quenching constant, τ_0 is the fluorophore average fluorescence lifetime, [Q] is the total quencher concentration and K_{SV} is the Stern-Volmer quenching constant. If the quenching process is solely due to diffusive encounters (collisions) between the compound and albumin, then K_{SV} is the dynamic constant (K_D) and k_q should have a value lower than the limiting diffusion rate in water (3×10^{10} M^{-1} s^{-1}) [4]. The obtained Stern-Volmer constants are 1.7×10^5 M^{-1} for **[ZnL(AcO)]** and 2.6×10^5 M^{-1} for **[ZnL$_2$]**. Since the Trp emission in albumins has τ_0 in the nanoseconds range, this means that the calculated k_q are above the collisional limit and, therefore, the quenching process has a static component, from the formation of a non-fluorescent ground complex between the compounds and albumin.

When small molecules bind to BSA, the association process can be analysed with the Scatchard equation [5]:

$$\log[(I_0 - I)/I] = \log K + n\ \log[Q]_f$$

where I_0 and I have the same meaning as in the Stern-Volmer equation, *K* is the association constant between the two species, *n* is the number of binding sites and $[Q]_f$ is the concentration of the free quencher [6]. Application of this analysis to both systems yields an association constant of 5.0×10^4 M^{-1} for the 1:1 complex, while **[ZnL$_2$]** shows a constant more than an order of magnitude higher, $K = 6.4\times10^5$ M^{-1}. In both cases one binding site is obtained, with $n = 0.91$ for **[ZnL(AcO)]** and 1.04 for **[ZnL$_2$]**. These results are in good agreement with previously published data for 8-hydroxyquinoline derivatives [7], and other metal complexes bearing hydrazone ligands [8]. Association constants in the range 10^4 – 10^5 M^{-1} are considered to represent moderate to strong binders, which may be delivered by the protein to their biological targets.

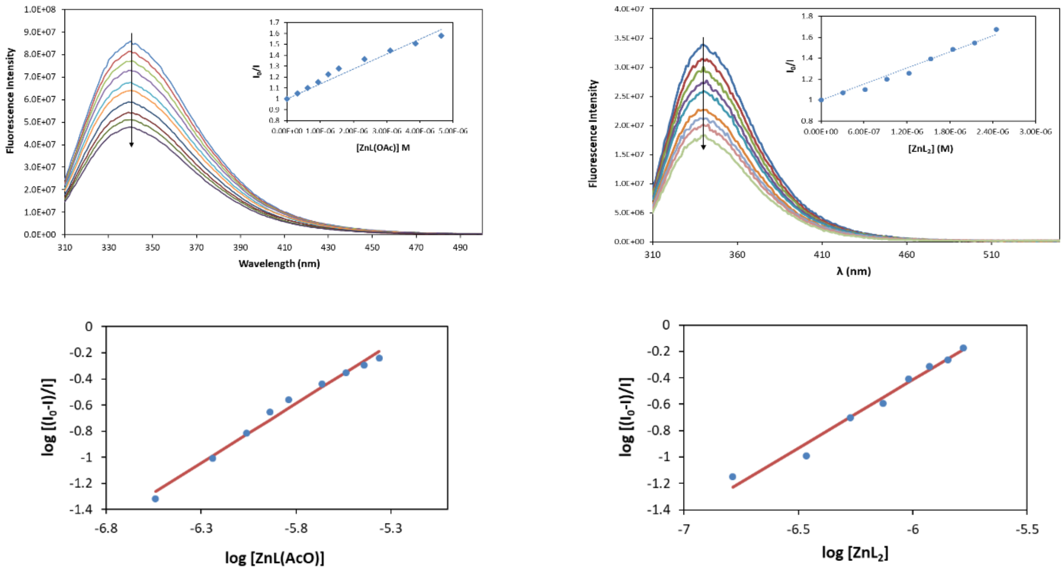

Figure S6. Top: Fluorescence emission of BSA (1.6 μM) in the presence of **[ZnL(AcO)]** (0 – 1.6 μM) and in the presence of **[ZnL₂]** (0 – 2.5 μM) with the Stern-Volmer plots as insets. Down: Scatchard plots corresponding to the above titrations.

Another useful spectroscopic technique to study these interactions is circular dichroism (CD) that may be used to detect protein conformational changes or the development of induced CD signals [9,10]. Although limited by its low sensitivity and requirement of relatively high amounts of chiral species, induced circular dichroism (ICD) signals provide valuable information on the interaction process. BSA is chiral but does not possess absorption bands above 300 nm (it is a non-absorbing host in this spectral range), while the complexes present absorption bands in the region 310 – 600 nm but they are the non-chiral (absorbing guest molecules). Upon binding, the symmetry of the non-chiral guest is perturbed by the chiral environment of the host resulting in the generation of non-zero rotational strengths [11]. Titrations of a fixed concentration of BSA (24 μM) with increasing amounts of each complex, revealed the appearance of ICD signals providing once more proof of the interaction between the complexes and BSA.

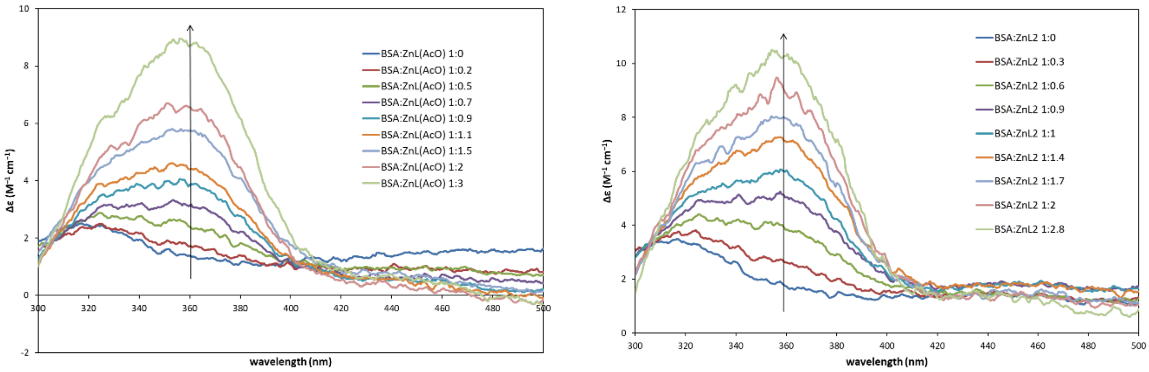

Figure S7. Titration of BSA (24 μM) with **[ZnL(AcO)]** (0 – 74 μM) and with **[ZnL₂]** (0 – 68 μM) followed by circular dichroism spectroscopy showing the appearance of ICD bands in the absorption region of the complexes. The experiments were done in PBS buffer, with a maximum content of 2% (v/v) DMSO; the induced CD spectra were obtained by subtracting the BSA spectrum measured under the same conditions.

S3 - Studies of interactions with ctDNA

The evaluation of the interaction of each complex with the genetic material (DNA) was also done using spectroscopic techniques, as used for BSA. However, no induced circular dichroism bands were observed, and the complexes did not show ability to displace the classical intercalator ethidium bromide (Figure S8). Additionally, the very poor solubility in water precluded the use of UV-Vis absorption titrations. Overall, the set of results obtained wit DNA makes us believe in a cell death mechanism that does not include a direct interaction with the genetic material.

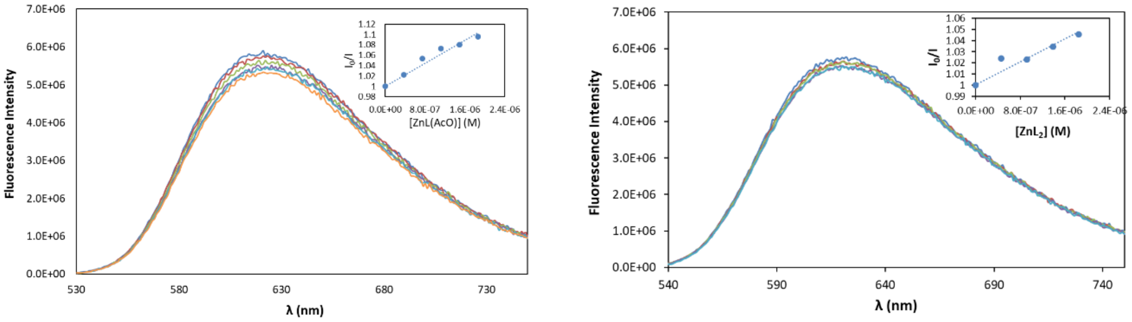

Figure S8. Titration of the system EB:DNA (1.85 μM: 2.27 μM) with **[ZnL(AcO)]** (0 – 3.7 μM, left) and with **[ZnL_2]** (0 – 2.3 μM, right) followed by fluorescence emission spectroscopy, using λ_{ex}= 510 nm and slits 5/5 nm. The experiments were done in HEPES buffer, pH 7.4 (0.1 M HEPES; 0.15 M KCl), with a maximum content of 0.8% DMSO (p/v).

S4 – Cell death and cell cycle analysis

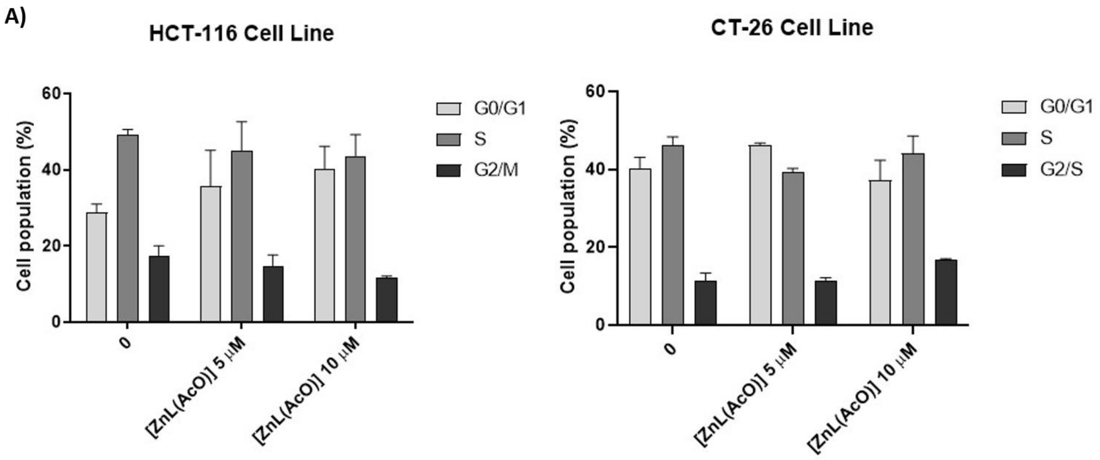

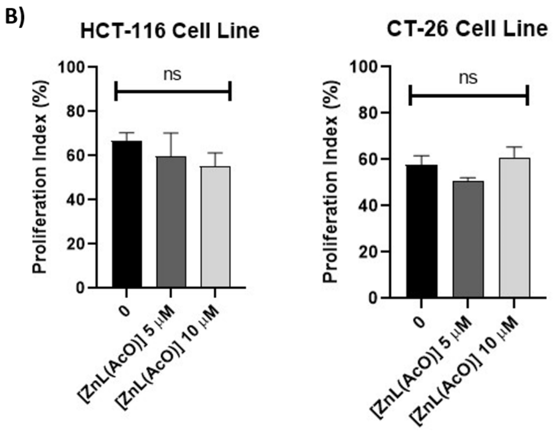

Figure S9. A) Percentage of HCT-116 and CT-26 cells cultured in a 2D setting in G0/G1, S, and G2/M cell cycle phases after a 24 h incubation with then Zn-complexes at 5 and 10 μM and with complete medium (0), obtained by flow cytometry analysis. **B)** Proliferation indexes for the complexes determined according to cell cycle distribution of exponentially growing populations of HCT-116 and CT-26 as the sum of percentage of cells in S and G2/M phases. Results correspond to the cell population expressed as mean percentage (%) ± SD of three independent experiments. Ns: non-significant.

Reference

1. Lakowicz, J. R. *Principles of Fluorescence Spectroscopy*, Springer: New York, NY, USA, 2007.
2. Coutinho, A.; Prieto, M. Ribonuclease-t(1) and alcohol-dehydrogenase fluorescence quenching by acrylamide—A laboratory experiment for undergraduate students. *J. Chem. Educ.* **1993**, *70*, 425–428.
3. Marquês, J.T.; de Almeida, R.F.M. Application of Ratiometric Measurements and Microplate Fluorimetry to Protein Denaturation: An Experiment for Analytical and Biochemistry Students. *J. Chem. Educ.* **2013**, *90*, 1522–1527.
4. Ware, W.R. Oxygen Quenching of Fluorescence in Solution: An Experimental Study of the Diffusion Process. *J. Phys. Chem.* **1962**, *66*, 455-458.
5. Scatchard, G. The Attractions of Proteins for Small Molecules and Ions. *Ann. New York Acad. Sci.* **1949**, *51*, 660–672.
6. Macii, F.; Biver, T. Spectrofluorimetric analysis of the binding of a target molecule to serum albumin: Tricky aspects and tips. *J. Inorg. Biochem.* **2021**, *216*, 111305.
7. Phopin, K.; Ruankham, W.; Prachayasittikul, S.; Prachayasittikul, V.; Tantimongcolwat, T. Insight into the Molecular Interaction of Cloxyquin (5-chloro-8-hydroxyquinoline) with Bovine Serum Albumin: Biophysical Analysis and Computational Simulation. *Int. J. Mol. Sci.* **2020**, *21*, 249. https://doi.org/10.3390/ijms21010249.
8. Sathyadevi, P.; Krishnamoorthy, P.; Jayanthi, E.; Butorac, R.R.; Cowley, A.H.; Dharmaraj, N. Studies on the effect of metal ions of hydrazone complexes on interaction with nucleic acids, bovine serum albumin and antioxidant properties. *Inorg. Chim. Acta* **2012**, *384*, 83–96.
9. Tedesco, D.; Bertucci, C. Induced circular dichroism as a tool to investigate the binding of drugs to carrier proteins: Classic approaches and new trends. *J. Pharm. Biomed. Anal.* **2015**, *113*, 34–42.
10. Pessoa, J.C.; Correia, I.; Gonçalves, G.; Tomaz, I. Circular dichroism in coordination compounds. *J. Argent. Chem. Soc.* **2009**, *97*, 151–165.
11. Allenmark, S. Induced circular dichroism by chiral molecular interaction. *Chirality* **2003**, *15*, 409-422.
